# Male and Female Tortricid Moth Response to Non-Pheromonal Semiochemicals

**DOI:** 10.3390/insects14110884

**Published:** 2023-11-16

**Authors:** Ajay P. Giri, Brent D. Short, Jaime C. Piñero

**Affiliations:** 1 Stockbridge School of Agriculture, University of Massachusetts, Amherst, MA 01003, USA; agiri@umass.edu; 2 Trécé Inc., Adair, OK 74330, USA; bshort@trece.com

**Keywords:** behavior, monitoring, semiochemical, IPM

## Abstract

**Simple Summary:**

In eastern North America, apple production faces threats from various moth species, particularly the codling moth (CM), Oriental fruit moth (OFM), redbanded leafroller (RBLR), obliquebanded leafroller (OBLR), and three-lined leafroller (TLLR) in the Tortricidae family. A study in Massachusetts orchards over two years examined the response of these moths to different lures, including commercial ones like Megalure. The results revealed Megalure’s attractiveness to both sexes of the OFM and CM. The addition of benzaldehyde enhanced the capture of male OFMs, suggesting its potential to improve commercial lures with aromatic compounds.

**Abstract:**

In eastern North America, apple orchards are often attacked by several species of tortricid moths (Lepidoptera), including *Cydia pomonella*, *Grapholita molesta*, *Argyrotaenia velutinana*, and *Pandemis limitata*. Sex pheromones are routinely used to monitor male moth populations. Adding plant volatiles to monitoring traps could increase the capture of moths of both sexes and improve the effectiveness of mating disruption systems. This study sought to quantify the attraction of adults of four tortricid moth species to five olfactory treatments, namely (1) Pherocon^®^ CM L2-P, (2) Pherocon Megalure CM 4K Dual^®^ (=Megalure), (3) Megalure + benzaldehyde, (4) TRE 2266 (linalool oxide + (*E*)-4,8-dimethyl-1,3,7-nonatriene (DMNT)), and (5) TRE 2267 (linalool oxide + DMNT + benzaldehyde), in non-mating disrupted commercial apple orchards in Massachusetts. The commercial lure Megalure was attractive to both sexes of *G. molesta* and *C. pomonella*. The addition of benzaldehyde to TRE 2266 or to Megalure significantly increased the capture of male *G. molesta* during the mid and late season of 2021. Only when benzaldehyde was added to TRE 2266 did the latter lure attract *P. limitata* in 2020 and 2021. The greatest number of tortricid moths (all four species combined) was captured by TRE 2267. This finding highlights the opportunity to enhance the attractiveness of a commercial lure through the addition of benzaldehyde, an aromatic compound, to Megalure. The potential of these additional volatiles to detect moths in a mating-disrupted orchard and/or remove female moths as a component of a management system is discussed.

## 1. Introduction

In eastern North America, apple *Malus domestica* (Borkh) is often attacked by several moth species in the family Tortricidae (Lepidoptera). Examples of tortricid pests include the codling moth *Cydia pomonella* (L.) (CM), the Oriental fruit moth *Grapholita molesta* (Busck) (OFM), and leafrollers such as the redbanded leafroller *Argyrotaenia velutinana* (Walker) (RBLR), obliquebanded leafroller *Choristoneura rosaceana* (Harris) (OBLR), and three-lined leafroller *Pandemis limitata* (Robinson) (TLLR) [1,2]. The larvae of tortricid moth species can feed on leaves, shoots, buds, and fruits. Fruit feeding can be categorized as external or internal. External feeders include the RBLR, TLLR, and OBLR, species in which the larvae cause the rolling of the leaves and feed on the surface of fruit [3]. The OFM and CM are internal feeders that bore into fruit; in the case of the CM, the larvae move directly into the seed cavity where they begin feeding on seeds [4].

The effective monitoring of tortricid moths is a centerpiece of integrated pest management programs. The most common strategy used by growers to control tortricid moths involves the use of timely sprays of insecticides [5,6]. However, the application of synthetic insecticides can be detrimental to the environment and to non-target species, and there is growing evidence of pest resistance to various types of insecticides [7,8,9]. Sex pheromone lures are widely used in traps to track the seasonal population dynamics of males of the CM, OFM, RBLR, TLLR, and OBLR in both conventional settings and orchards under mating disruption [10]. For mating disruption to be effective, areas larger than 2.5 ha are recommended [8]. Thus, this control measure is more suitable for larger-scale conventional growers [11,12]; meanwhile, for small-scale growers, this control method is logistically unsuitable and expensive. Because of the high concentration of pheromones used for mating disruption, traps loaded with standard lures are not effective and specialized lures are needed [13]. In addition, there is a chance in mating- and non-mating-disrupted orchards that fruit infestation by mated females immigrating from outside areas will occur. Monitoring female moth populations may allow for more precise action thresholds and improved moth monitoring under conventional and/or mating-disrupted blocks [6,14,15]. Therefore, there is a need to identify alternative lures that can attract female moths [16,17].

It is well known that CM and OFM adults are attracted to hostplant odors [18,19,20,21,22,23,24,25,26]. The identification of pear ester (ethyl (*E*,*Z*)-2,4-decadienoate) by Light et al. [16] as a compound attractive to the CM improved the ability of codlemone ((*E*,*E*)-8,10-dodecadien-1-ol), the synthetic CM sex pheromone, to detect males in the Western USA. Furthermore, the combination of these two attractants increased the capture of female CMs [8,27]. Similarly, the addition of acetic acid to pear ester and/or (*E*)-4,8-dimethyl-1,3,7-nonatriene (DMNT) to odor mixtures resulted in blends that improved female CM capture [28,29]. Most recently, Knight et al. [6] evaluated pyranoid (PyrLOX) and furanoid (FurLOX) linalool oxide, floral volatiles produced by the oxidation of linalool, as additional attractants in the synergistic blend of pear ester, acetic acid, and DMNT. Only the addition of PyrLOX, 6-ethenyl-2,2,6-trimethyloxan-3-ol, to the blend of pear ester, acetic acid, and DMNT attracted significantly more male and female CMs than other non-pheromone and pheromone blends tested. In the case of the OFM, blends of volatile compounds have been found to be particularly attractive. For example, Piñero and Dorn [23] reported a bioactive five-compound mixture of three green leaf volatiles ((*Z*)-3-hexen-1-ol, (*E*)-2-hexanal, and (*Z*)-3-hexen-1-yl acetate) and two aromatic compounds (benzaldehyde and benzonitrile), which acted synergistically as female OFM attractants. Il’ichev et al. [24] reported that three compounds, (*Z*)-3-hexenyl acetate, (*E*)-*β*-ocimene and (*E*)-*β*-farnesene, in a 1:2:2 ratio attracted most male OFMs, but not females, when tested in the field. In turn, Lu et al. [30] reported a mixture of eight compounds: 1-hexanol, nonanal, ethyl butanoate, butyl acetate, ethyl hexanoate, hexyl acetate, hexyl butanoate, and farnesene; this was formulated in a 1:1:100:70:7:5:1:4 ratio that attracted both sexes of the OFM in the field. In a field study conducted in Chile, Barros-Parada et al. [31] evaluated the three blends reported by Piñero and Dorn [23], Il’chev et al. [24], and Lu et al. [30], and found that the five-compound mixture produced by Piñero and Dorn [23] was significantly more attractive to male OFMs than the other blends. Knight et al. [32] reported that the addition of (*E*)-*β*-ocimene, or (*E*)-*β*-farnesene, or butyl hexanoate septa lures to aqueous terpinyl acetate plus brown sugar (TAS) significantly increased the total OFM captures in Ajar traps. Other studies (e.g., [33,34,35,36,37]) have evaluated combinations of the OFM sex pheromone and kairomones, and improved trap designs with variable results in trap both sexes of the OFM.

For leafrollers, a number of plant volatiles, including (*E*,*Z*)-2,4-decadienoate (pear ester), butyl hexanoate, (*E*)-*β*-farnesene, (*E*)-*β*-ocimene, (*E*)-4,8-dimethyl-1,3,7-nonatriene, (*Z*)-3-hexenyl acetate (leaf acetate) and farnesol, have been tested alone or in combination with glacial acetic acid (GAA) and codlemone [34]. Interestingly, traps baited with codlemone (sex pheromone of CM), pear ester, and GAA co-lure have caught both sexes of the OBLR, along with other tortricids (the CM, OFM, *Pandemis pyrusana* (Kearfott)). However, none of the above-mentioned plant volatiles have significantly increased the total OBLR captures compared to the OBLR sex pheromone. The attraction of male and female OBLRs [38,39], in addition to male and female TLLRs [40], to the blend of 2-phenyl ethanol and acetic acid has been reported. Furthermore, this binary blend of kairomones was found to attract more OBLR adults in mating-disrupted orchards than in pheromone-baited traps [38].

Mass trapping could be another desirable use of lures that are attractive to female moths. For instance, the four-kairomone blend that includes pear ester, acetic acid, DMNT, and PyrLOX was found to be an effective “female removal” strategy for CM management in the western region of the USA (e.g., Washington State, Oregon State) [41]. However, CM populations from different geographical regions seem to have variable responses to the 4K lure (also known as Megalure), as shown in studies conducted in Italy [41,42,43,44,45,46,47].

This study was designed to quantify the response of males and females of multiple tortricid moth species (CM, OFM, RBLR and TLLR) in Massachusetts apple orchards to combinations of commercially available (Pherocon^®^ CM L2-P (Adair, OK, USA), and Megalure CM 4K Dual^®^ (Adair, OK, USA)) and benzaldehyde experimental lures. Benzaldehyde is one of the most abundant aromatics in the headspace of peach shoots [21]. We hypothesized that, when added to different blends of kairomones, benzaldehyde would enhance moth responses.

## 2. Materials and Methods

### 2.1. Study Sites

Field-scale studies were conducted over a two-year period (2020–2021) in non-mating-disrupted commercial apple orchards in Massachusetts that received no insecticides targeting tortricid moths. Insecticides were applied against key pests such as plum curculio, *Conotrachelus nenuphar* (Herbst) (Coleoptera: Curculionidae), earlier in the season and apple maggot fly, *Rhagoletis pomonella* (Walsh) (Diptera: Tephritidae), during July and August. The insecticides applied against the first pest included the organophosphate phosmet (Imidan 70-W^®^; Gowan Co., Yuma, AZ, USA), the neonicotinoid thiacloprid (Calypso^®^, Bayer Crop Science LP, Monheim am Rhein, Germany) and the oxadiazine indoxacarb (Avaunt^®^, FMC, Philadelphia, PA, USA) at recommended rates. Meanwhile, the insecticides used against *R. pomonella* were Imidan 70-W^®^, Avaunt^®^, and the neonicotinoid acetamiprid (Assail 30G^®^, United Phosphorus Inc., King of Prusia, PA, USA). Fungicides to control scab and other summer diseases were applied as deemed necessary by the growers.

The 2020 study was carried out from 13 May to 18 September at Sholan Farms, in Leominster, MA, USA, and the size of the experimental block was 3 ha. (Supplementary Figure S1). The 2021 study was carried out from 10 June to 9 September, in four commercial apple orchards: Sholan Farms (Leominster, MA, USA), Sentinel Farm (Belchertown, MA, USA), Honeypot Hill Orchards (Stow, MA, USA), and at the University of Massachusetts (UMass) Agricultural Learning Center (Amherst, MA, USA), for a combined experimental area of 4.5 ha (Supplementary Figure S2). The apple trees in all the orchards ranged from small (M.26; approx. 1000 per acre) to medium (M.7; approx. 140 per acre) in size. The cultivars most commonly present in the test blocks were McIntosh, Empire, Honeycrisp, Fuji, Gala, Ginger Gold, and Cortland. Except for the UMass Agricultural Learning Center, which is certified organic and has received selected OMRI-listed materials, all other orchards received standard insecticide spray regimes from the growers, as mentioned above in the first study.

### 2.2. Odor Treatments and Trap Types

For both studies, the same five proprietary lures were evaluated: (1) Pherocon^®^ CM L2-P (CM L2-P), (2) Megalure CM 4K Dual^®^ (hereafter referred to as Megalure), (3) Megalure + benzaldehyde, (4) TRE 2266 (=linalool oxide + DMNT, and (5) TRE 2267 (=linalool oxide + DMNT + benzaldehyde) (Table 1) (Trécé Inc., Adair, OK, USA). Benzaldehyde is a plant volatile that, when present in a mixture, has shown to be attractive to female OFMs [23,48]. Unbaited traps served as negative controls.

All lures were placed inside orange-colored delta-shaped traps (Pherocon^®^ VI, Trécé Inc., Adair, OK, USA) containing liners coated with cold-melt adhesive [49]. All experimental lures were formulated by Trécé in a black polyvinyl chloride (PVC) proprietary matrix except for one component of Megalure, acetic acid, which was formulated in a white membrane-based cup. Each treatment was replicated 8 and 10 times in 2020 and 2021, respectively. Traps were spaced at 15 m intervals along the perimeter of apple blocks and were suspended from the upper third of the tree canopy. During trap set up, the relative location of each treatment was randomized within a replicate. Traps were rotated weekly clockwise within a replication to minimize the effect of position. All lures and sticky liners were replaced at 4-week intervals.

### 2.3. Data Collection

For the 2020 study, traps were examined weekly from 19 May until 18 September. For the 2021 study, traps were examined weekly from 16 June to 9 September. The 2021 study missed the early season trapping period due to a delay in the procurement of lures. All adult moths captured were identified according to species and were placed in 25 mL glass vials containing 70% ethanol. The sex of each moth species was identified according to Fuková et al. [50] and Shang et al. [51] by examining the genitalia under a dissecting microscope (Supplementary Figure S3).

### 2.4. Weather Information

The mean weekly temperature (°C), total weekly precipitation (mm), and degree day (DD) accumulation information (Supplementary Figure S4) throughout the various trapping periods in 2020 and 2021 was obtained from Sholan Farms (Leominster, MA, USA) using the NEWA website. No extreme weather events occurred during either trapping year.

### 2.5. Statistical Analysis

A preliminary analysis involving Repeated Measures Analysis of Variance (ANOVA) using trap-capture data (males only) separately for each moth species revealed a significant interaction between treatment and week, indicating differential responses by the male moths to the treatments over time. Based on those preliminary analyses, moth captures in 2020 were divided into three seasonal time periods (early season: 13 May to 19 June; mid-season: 20 June to 4 August; and late season: 5 August to 18 September). A Repeated Measures ANOVA conducted on 2021 data revealed similar interactions. As a result, the trap capture dates were divided into two seasonal time periods (mid-season: 10 June to 21 July, and late season: 22 July to 9 September). Early-season data were not collected in 2021 due to logistical issues. For each year, the trap-capture data from each period were explored using generalized linear mixed models, assuming a Poisson distribution to assess the effects of ‘treatment’ (fixed effect) and ‘block’ (random factor), and the two-way interactions among them. Analyses of Variance (ANOVAS) were conducted for each moth species and trapping period using data transformed to (x + 0.5)^1/2^, prior to conducting an analysis to stabilize variances. Whenever appropriate, the means were separated using the post hoc Tukey-protected HSD test at a 5% probability level. All statistical analyses were performed using STATISTICA v.13 (TIBCO Software Inc., Palo Alto, CA, USA). Female moth captures were not analyzed statistically because of insufficient numbers.

## 3. Results

### 3.1. 2020 Study

#### 3.1.1. Early Season Captures (13 May to 19 June)

**Figure 1:**
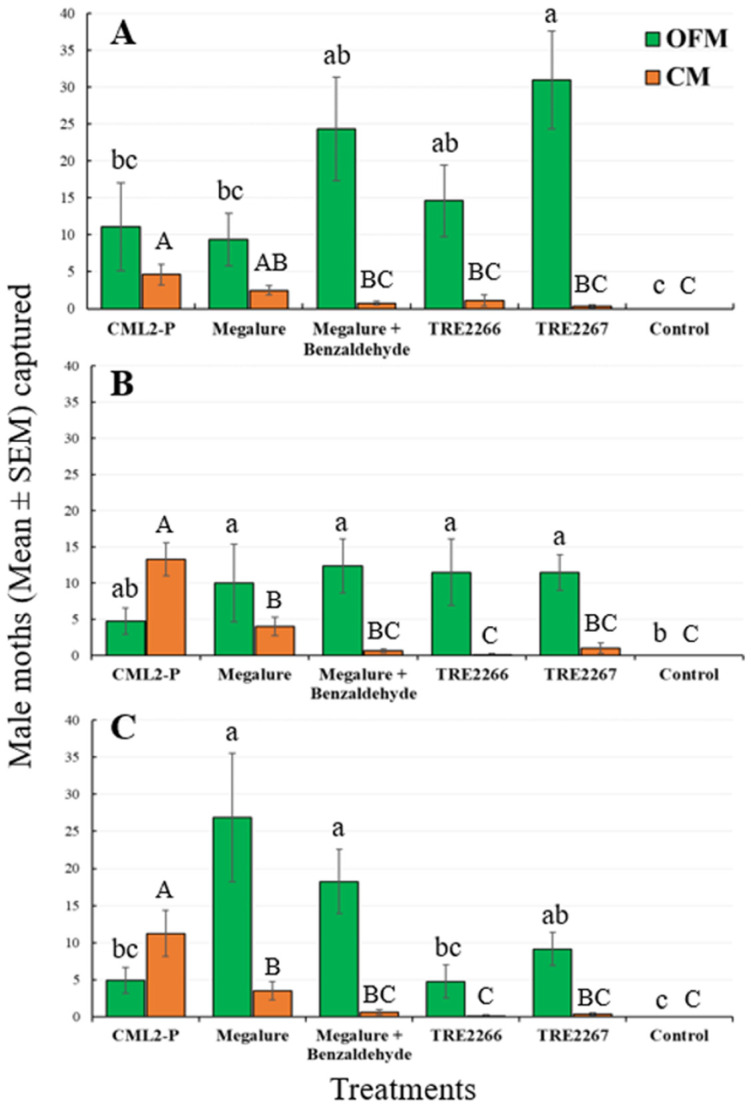
Captures (mean ± SEM) of OFM and CM males in delta traps baited with five olfactory treatments and non-baited traps in the (**A**) early season, (**B**) mid-season, and (**C**) late season in 2020. TRE 2266 = linalool oxide and (*E*)-4,8-dimethyl-1,3,7-nonatriene (DMNT); TRE 2267 = TRE 2266 + benzaldehyde. For each moth species, bars superscribed with the same letter do not differ significantly among treatments (Tukey-protected HSD tests as *p* = 0.05).

During this period, the mixed-model analyses revealed that the treatment had a significant effect on the capture of males of four species of tortricids: OFMs (ANOVA F_5,35_ = 15.3, *p* < 0.001), CMs (ANOVA F_5,35_ = 10.3, *p* < 0.001), RBLRs (ANOVA F_5,35_ = 30.4, *p* < 0.001), and TLLRs (ANOVA F_5,35_ = 3.9, *p* < 0.006). The proprietary experimental benzaldehyde-containing lure TRE 2267 caught significantly more male OFMs than the CM L2-P and Megalure lures (Figure 1A).

**Figure 2:**
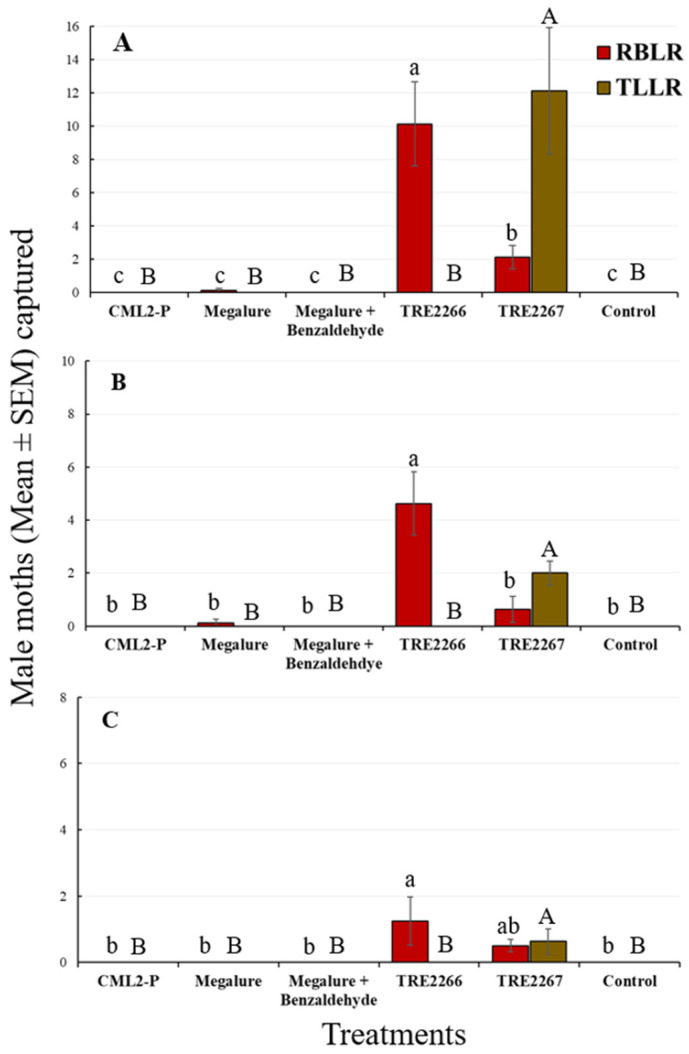
Captures (mean ± SEM) of RBLR and TLLR males in delta traps baited with five olfactory treatments and non-baited traps in the (**A**) early season, (**B**) mid-season, and (**C**) late season in 2020. TRE 2266 = linalool oxide and (*E*)-4,8-dimethyl-1,3,7-nonatriene (DMNT); TRE 2267 = TRE 2266 + benzaldehyde. For each moth species, bars superscribed with the same letter do not differ significantly among treatments (Tukey-protected HSD tests at *p* = 0.05).

The mean number of male OFMs captured in Megalure, Megalure + benzaldehyde, and TRE 2266 was statistically similar to the pheromone lure CM L2-P. Traps baited with Megalure captured, on average, 1.13 female OFMs per trap during this period (Table 2). In terms of CM captures, CM L2-P captured significantly more males than any other lure, and this treatment was statistically similar to Megalure (Figure 1A). Captures of male CMs in traps baited with Megalure, Megalure + benzaldehyde, TRE 2266, and TRE 2267 were not statistically different. No female CMs were captured in this period. As for male RBLRs, traps baited with TRE 2266 captured significantly more moths than any other treatment (Figure 2A). The addition of benzaldehyde to TRE 2266 (=TRE 2267) resulted in a significant reduction in the number of male RBLRs captured by traps. Notably, traps baited with TRE 2267 captured comparatively high numbers of male TLLRs, which were completely absent in any other treatment. None of the lures were attractive to RBLR and TLLR females.

#### 3.1.2. Mid-Season Captures (20 June to 4 August)

There was a significant effect of treatment for male OFMs (ANOVA F_5,35_ = 7.4, *p* < 0.001), male CMs (ANOVA F_5,35_ = 23.4, *p* < 0.001), and male RBLRs (ANOVA F_5,35_ = 17.7, *p* < 0.001). During this period, there were no significant differences in male OFM captures in traps baited with kairomone-only lures (Megalure, Megalure + benzaldehyde, TRE 2266, and TRE 2267) and the pheromone lure (Figure 1B). Traps baited with Megalure + benzaldehyde captured 0.13 female OFMs on average (Table 2). CM L2-P was the most attractive lure for male CMs (Figure 1B), followed by Megalure, Megalure + benzaldehyde, and TRE 2267, all of which were statistically similar. On average, traps baited with Megalure captured one female CM per trap during this period, followed by Megalure + benzaldehyde (0.25 CM, on average) and TRE 2266 (0.13 CM, on average) (Table 2). For male RBLRs, TRE 2266 was significantly the most attractive lure (Figure 2B). As observed earlier in the season, only the TRE 2267 lure attracted male TLLRs (Figure 2B), and none of the lures attracted RBLR and TLLR females.

#### 3.1.3. Late Season Captures (5 August to 18 September)

There was a significant effect of treatment on the capture of male OFMs (ANOVA F_5,35_ = 21.7, *p* < 0.001), male CMs (ANOVA F_5,35_ = 17.4, *p* < 0.001), and male RBLRs (ANOVA F_5,35_ = 3.9, *p* < 0.001). Megalure and Megalure + benzaldehyde attracted significantly more male OFMs than TRE 2266 and CM L2-P (Figure 1C). Male OFM captures in traps baited with TRE 2266, TRE 2267, and CM L2-P were statistically similar. There were very few female OFMs (five total) and female CMs (two total) captured in this period (Table 2). CM L2-P was significantly more attractive to male CMs than other lures, and TRE 2266 and TRE 2267 lures were attractive to male RBLRs. Male TTLRs were captured exclusively in traps baited with TRE 2267 (Figure 2C).

### 3.2. 2021 Study

#### 3.2.1. Mid-Season Captures (10 June to 21 July)

**Figure 3:**
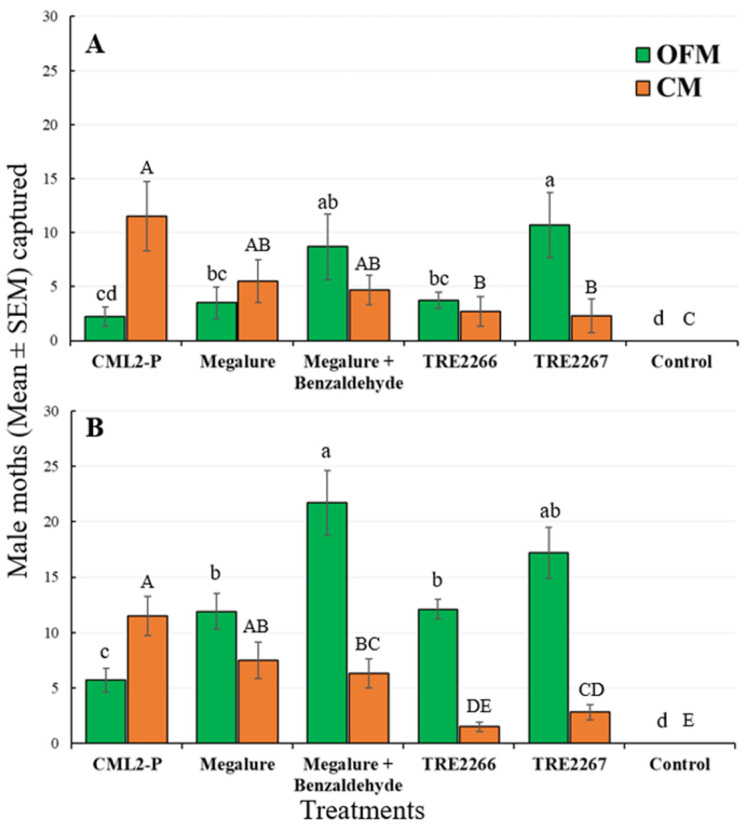
Captures (mean ± SEM) of OFM and CM males in delta traps baited with five olfactory treatments and non-baited trap in the (**A**) mid-season and (**B**) late season in 2021. TRE 2266 = linalool oxide and (*E*)-4,8-dimethyl-1,3,7-nonatriene (DMNT); TRE 2267 = TRE 2266 + benzaldehyde. For each moth species, bars superscribed with the same letter do not differ significantly among treatments (Tukey-protected HSD test as *p* = 0.05).

**Figure 4:**
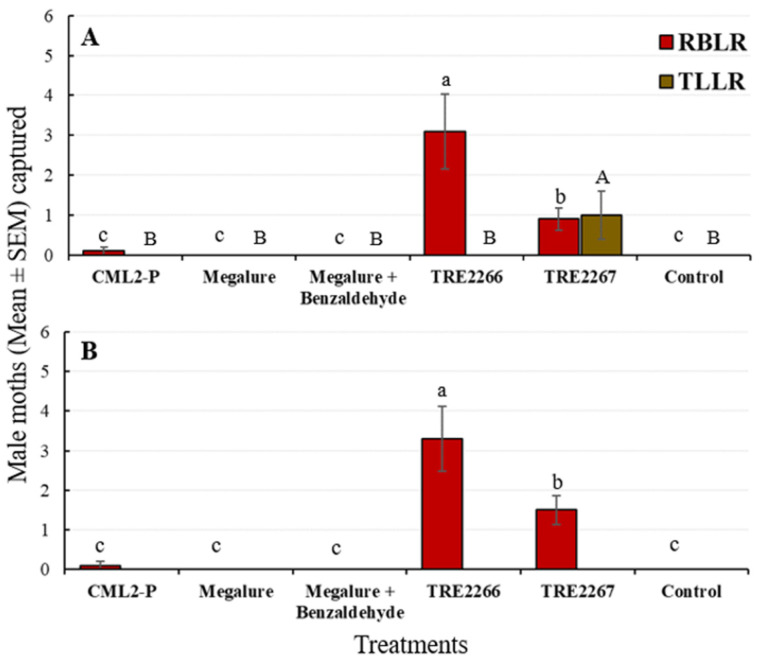
Captures (mean ± SEM) of RBLR and TLLR males in delta traps baited with five olfactory treatments and non-baited traps in the (**A**) mid-season and (**B**) late season in 2021. TRE 2266 = linalool oxide and (*E*)-4,8-dimethyl-1,3,7-nonatriene (DMNT); TRE 2267 = TRE 2266 + benzaldehyde. For each moth species, bars superscribed with the same letter do not differ significantly among treatments (Tukey-protected HSD test at *p* = 0.05).

During this period, there was a significant effect of treatment on male OFMs (ANOVA F_5,45_ = 10.4, *p* < 0.001), male CMs (ANOVA F_5,45_ = 53.6, *p* < 0.001), male RBLRs (ANOVA F_5,45_ = 16.5, *p* < 0.001), and male TLLRs (ANOVA F_5,45_ = 3.9, *p* < 0.005). TRE 2267 attracted significantly more male OFMs than any other lures except Megalure + benzaldehyde (Figure 3A). Male OFM captures did not differ statistically among traps baited with Megalure, Megalure + benzaldehyde, and TRE 2266. Only traps baited with Megalure were attractive to female OFMs, although very low numbers of females were captured by traps during the mid-season (Table 2). For male CMs, there were no significant differences in captures using CM L2-P, Megalure, and Megalure + benzaldehyde. On average, traps baited with Megalure + benzaldehyde captured 2.2 female CMs, whereas traps baited with Megalure alone captured 1.4 female CMs and those baited with TRE 2267 caught 0.3 female CMs (Table 2). No other treatment attracted female CMs. TRE 2266 was the most attractive lure for male RBLRs, followed by TRE 2267, and male TLLRs were only attracted to TRE 2267 (Figure 4A).

#### 3.2.2. Late-Season Captures (22 July to 9 September)

Significant differences among treatments were recorded for male OFMs (ANOVA F_5,45_ = 53.6, *p* < 0.001), male CMs (ANOVA F_5,45_ = 38.3, *p* < 0.001), and male RBLRs (ANOVA F_5,45_ = 20.6, *p* < 0.001). Megalure + benzaldehyde attracted significantly more male OFMs than CM L2-P, Megalure, and TRE 2266 (Figure 3B). The captures of male OFMs were statistically similar among traps baited with Megalure, TRE 2266, and TRE 2267. On average, Megalure + benzaldehyde attracted 1.2 female OFMs, followed by 0.7 in Megalure, 0.4 in TRE 2267, and 0.1 in TRE 2266 (Table 2). In terms of CM captures, CM L2-P attracted the most males (Figure 3B). On average, Megalure attracted the highest number (1.6) of female CMs (Table 2). TRE 2267 attracted RBLRs in this period; however, TRE 2266 was the most attractive lure for RBLRs (Figure 4B).

## 4. Discussion

In the Northeastern region of the USA, historically, there has been a limited amount of research involving evaluations of semiochemicals with tortricid moths. This is an important consideration given that the response of moth species has been shown to vary across different regions. For instance, CM adults have shown variable responses to kairomone lures, including pear ester and, most recently, the 4K blend that has the same components as Megalure [41,42,43,44,45,46,47]. Factors potentially influencing the varied response documented across regions include differences in weather and different strains or ecotypes of moths, which may differ with respect to host preference, mobility, fitness, reproductive capacity, seasonal development, and pesticide resistance [45,52,53,54,55]. The present study was designed to test different kairomone blends in orchards not subjected to mating disruption under New England conditions.

To our knowledge, this study represents the first field report of a strong OFM attraction to Pherocon Megalure CM 4K Dual^®^, a non-pheromonal blend comprising four kairomones, and the effects of adding benzaldehyde to Megalure and to a mixture of linalool oxide and DMNT. TRE 2266 is a reduced version of the commercial 4K lure Megalure (pear ester, acetic acid, DMNT, linalool oxide) to make a 2K blend (DMNT and linallol oxide). Originally, our study was designed to see what effect DMNT and linalool oxide would have on moth captures in the absence of acetic acid and pear ester, given that pear ester alone and acetic acid alone can elicit the response of CMs and OFMs, as shown by many studies. Surprisingly, in this study, DMNT + linalool was attractive to leafrollers and to TLLRs when benzaldehyde was added. Benzaldehyde is an aromatic compound that is present in many plant species, but that is largely present in the family Rosaceae [56,57]. Previously, Natale et al. [21] reported OFM attraction to benzaldehyde when in a mixture containing (*Z*)-3-hexen-1-yl acetate and (*Z*)-2-hexene-1-ol. In turn, Piñero and Dorn [23] and Piñero et al. [48] documented that the combination of two aromatic compounds (benzaldehyde and benzonitrile) and three green leaf volatiles ((*Z*)-3-hexen-1-ol, (*E*)-2-hexenal, and (*Z*)-3-hexen-1-yl acetate) had a synergistic effect on female OFMs at the level of odor processing in the antennal lobes and attraction. In addition, other Lepidopteran insects, such as the cabbage looper moth, *Trichoplusia ni* (Hübner), the cabbage butterfly, *Pieris rapae* (Linnaeus), and the cotton bollworm, *Helicoverpa armigera* (Hübner), use benzaldehyde as a critical component of odor mixtures to locate hosts [58,59,60]. The application of benzaldehyde for semiochemical-based pest management has also been explored in the Coleopteran family Curculionidae. For instance, Piñero and Prokopy [61] reported the synergistic response of *Conotrachelus nenuphar* to grandisoic acid (a male-produced aggregation pheromone) when in combination with benzaldehyde. In another study, Lohonyai et al. [62] reported benzaldehyde as a suitable early-season monitoring tool for legume weevil, *Sitona humeralis* (Stephens). Most recently, Ethington et al. [63] reported the significant attraction of the peach bark beetle, *Phlorotribus liminaris* (Harris), to traps baited with benzaldehyde and its carrier ethanol.

Our combined results indicate that Megalure is attractive to both OFM and CM males, and has the potential to attract females of both species. Remarkably, the addition of benzaldehyde to TRE 2266 (a mixture of linalool oxide and DMNT), which resulted in TRE 2267, or to the Megalure lure significantly increased the capture of male OFMs during the mid-season and late season of 2021, relative to either TRE 2266 or Megalure alone. However, the low level of female moths captured across the experiment diminished our ability to adequately record the effects of treatment on female OFMs. Xiang et al. [64] reported that traps baited with benzaldehyde captured significantly more male OFMs when compared to octanal, nonanal and decanal, but did not capture significantly more than when using the OFM sex pheromone during testing in the field. In contrast, the addition of benzaldehyde to Megalure did not increase or decrease male or female CM capture. Interestingly, the addition of benzaldehyde to TRE 2266 (=TRE 2267) resulted in a material that was attractive to TLLRs, but that inhibited the trap capture of RBLRs. Previously, using a Y-tube olfactometer, Vallat and Dorn [65] reported the repellent effect of benzaldehyde to female CMs. Benzaldehyde is one of the major components of apple flower headspace [66], and its quantity decreases after petal fall. We postulate that benzaldehyde might be involved in the repellent effect the apple tree may have on CMs early in the season. Whether benzaldehyde may play a different role (attraction vs. repellency) in OFMs, CMs, RBLRs, and TLLRs as the season progresses remains to be elucidated.

In the present study, the CM pheromone lure (CM L2-P) was used as a positive control for CMs. Surprisingly, CM L2-P also attracted male OFMs, and the extent of response was comparable to that to Megalure during both the early and mid-season across the two study years. The documented male OFM response to CM L2-P echoes findings from a field study conducted in Argentina and Chile by Knight et al. [67], where the CM pheromone was as attractive to OFMs as a host plant volatile blend and acetic acid colure. Additionally, some studies have reported significant increases in male OFM captures in traps baited with the CM sex pheromone (codlemone) and OFM sex pheromone (a three-component blend of *Z*-8-dodecenyl acetate, *E*-8-dodecenyl acetate, and *Z*-8-dodecenol) [67,68,69]. Zhang et al. [70] provided insights at the molecular level by revealing a high level of identity between the pheromone-binding protein (PBP) in the CM (CpomPBP1) and OFM (Gmol1PBP1), suggesting a potential structural similarity. This shared structural feature may contribute to the observed synergistic response of the OFM to codlemone. We believe that such an unexpected alignment in the male OFM response to CM L2-P and Megalure, when coupled with shared PBP, underscores intriguing complexities in the interplay between pheromones and moth behavior, opening avenues for further exploration in the field.

The proportion of female moths captured by traps was very low compared to that of males. For instance, Megalure captured very low numbers of female moths (2.7% and 11.8% for OFMs and CMs, respectively) in the 2020 study. This pattern of results differs from studies by Knight et al. [6] in Washington State, who showed much higher levels of female CM attraction to Megalure (63–80% of the total number of moths captured were females). Additionally, Preti et al. [41] reported that Megalure attracted a greater number of female CMs in the Northwestern USA when compared to codlemone + acetic acid and a combination of codlemone, pear ester, DMNT, linalool oxide and acetic acid. It is not clear whether the comparatively lower number of females captured by Megalure-baited traps in Massachusetts found in the present study and in Italy might be due to differences in strains or the ecotypes of the moths, or to some other factors. Our results highlight the need to evaluate additional host plant volatile blends in combination with Megalure or its components to potentially increase female CM captures in specific regions.

When combining all four species that were present in the study sites, namely OFMs, CMs, RBLRs, and TLLRs, the greatest number of captures occurred in traps baited with TRE 2267, which contains benzaldehyde in its formulation. This study was originally designed to include the response of OBLRs, but no OBLRs were captured in the study sites. One application of our results is in the context of improved monitoring, particularly in orchards under mating disruption. For instance, benzaldehyde-containing materials can serve as multi-species lures that not only improve male OFM monitoring, but also facilitate the detection of less prevalent species such as the TLLR. The factors underlying the observed reduction in the capture of RBLRs in odor-baited traps when benzaldehyde was present are unknown.

## 5. Conclusions

This two-year study showed that the addition of benzaldehyde to Megalure significantly increased the response of male OFMs during the mid-season and late season of 2021. Male RBLRs responded strongly to linalool oxide + DMNT (=TRE 2266), whereas the same lure with benzaldehyde added was very attractive to TLLRs. Our combined findings can be used as baseline information to improve semiochemically based monitoring and control systems for multiple tortricid pests. Future research should evaluate the role that benzaldehyde at various release rates plays in tortricid moth attraction so that high-quality attractant signals can be presented to female moths, resulting in more reliable attraction under variable environmental conditions.

## Figures and Tables

**Table 1:** Types of lures used for the 2020 and 2021 field evaluations.

	Lure	Description
1	Pherocon^®^ CM L2-P	Codling moth pheromone lure (positive control)
2	Pherocon^®^ Megalure CM 4K Dual^®^	Pear ester, acetic acid, (*E*)-4,8-dimethyl-1,3,7-nonatriene (DMNT) and PyrLOX [3]
3	Pherocon^®^ Megalure CM 4K Dual^®^ + benzaldehyde	Blend of four kairomones + benzaldehyde
4	TRE 2266	Linalool oxide + DMNT
5	TRE 2267	TRE 2266 + benzaldehyde
6	Unbaited control	Negative control

**Table 2:** Average number of females captured, according to moth species (OFM and CM) and treatment, during the trapping periods in 2020 and 2021.

Lures	Average Number of Female Moths Captured per Season per Trap									
	2020 (*n* = 8)						2021 (*n* = 10)			
	OFM			CM			OFM		CM	
	Early	Mid	Late	Early	Mid	Late	Mid	Late	Mid	Late
CM L2-P	0	0	0	0	0	0	0	0	0	0
Megalure	1.13	0	0.13	0	1	0.13	0.2	0.7	1.4	1.6
Megalure + benzaldehyde	0.63	0.13	0.38	0	0.25	0	0.2	1.2	2.2	0.8
TRE 2266	0.13	0	0.13	0	0.13	0.13	0	0.1	0	0.4
TRE 2267	0.38	0	0	0	0	0	0	0.4	0.3	0.2

## Data Availability

The data presented in this study are available upon request from the corresponding author.

## Supplementary Materials

The following supporting information can be downloaded at: https://www.mdpi.com/article/10.3390/insects14110884/s1, Figure S1: Trap deployment along the perimeter of Sholan Farms in 2020; Figure S2: Trap deployment at (**A**) Sholan farms, (**B**) UMass Agricultural Learning Center, (**C**) Sentinel Farm, and (**D**) Honeypot Hill Orchards in 2021; Figure S3: Male genitalia (right) and female genitalia (left) of OFM (**A**,**B**), CM (**C**,**D**) and RBLR (**E**,**F**); Figure S4: Mean weekly captures of OFM (green bars) and CM (yellow bars) in early, mid, and late season during 2020 (**A**) and mid and late season during 2021 (**B**).

## References

[1] Chapman P.J., Lienk S.E. (1971). Tortricid Fauna of Apple in New York (Lepidoptera: Tortricidae).

[2] Lacey L.A., Arthurs S.P., Knight A.L., Huber J. (2007). Microbial control of lepidopteran pests of apple orchards. Field Manual of Techniques in Invertebrate Pathology: Application and Evaluation of Pathogens for Control of Insects and Other Invertebrate Pests.

[3] Knight A.L., Judd G.J., Gilligan T., Fuentes-Contreras E., Walker W.B. (2019). Integrated management of tortricid pests of tree fruit. Integrated Management of Diseases and Insect Pests of Tree Fruit.

[4] Williamson E.R., Folwell R.J., Knight A.L., Howell J.F. (1996). Economics of employing pheromones for mating disruption of the codling moth, *Carpocapsa pomonella*. Crop Prot..

[5] Knight A.L. (2008). Codling moth areawide integrated pest management. Areawide Pest Management: Theory and Implementation.

[6] Knight A.L., Mujica V., Herrera S.L., Tasin M. (2019). Addition of terpenoids to pear ester plus acetic acid increases catches of codling moth (Lepidoptera: Tortricidae). J. Appl. Entomol..

[7] Knight A.L., Brunner J.F., Alston D. (1994). Survey of azinphosmethyl resistance in codling moth (Lepidoptera: Tortricidae) in Washington and Utah. J. Econ. Entomol..

[8] Ioriatti C., Lucchi A. (2016). Semiochemical strategies for tortricid moth control in apple orchards and vineyards in Italy. J. Chem. Ecol..

[9] Wan F., Yin C., Tang R., Chen M., Wu Q., Huang C., Qian W., Rota-Stabelli O., Yang N., Wang S. (2019). A chromosome-level genome assembly of *Cydia pomonella* provides insights into chemical ecology and insecticide resistance. Nat. Commun..

[10] Gut L.J., Brunner J.F. (1996). Implementing codling moth mating disruption in Washington pome fruit orchards. Tree Fruit Research Extension Center Information Series.

[11] Akotsen-Mensah C., Blaauw B., Short B., Leskey T.C., Bergh J.C., Polk D., Nielsen A.L. (2020). Using IPM-CPR as a management program for apple orchards. J. Econ. Entomol..

[12] Orpet R.J., Jones V.P., Beers E.H., Reganold J.P., Goldberger J.R., Crowder D.W. (2020). Perceptions and outcomes of conventional vs. organic apple orchard management. Agric. Ecosyst. Environ..

[13] Knight A.L., Light D.M. (2005). Developing action thresholds for codling moth (Lepidoptera: Tortricidae) with pear ester-and codlemone-baited traps in apple orchards treated with sex pheromone mating disruption. Can. Entomol..

[14] Landolt P.J., Ohler B., Lo P., Cha D., Davis T.S., Suckling D.M., Brunner J. (2014). N-Butyl Sulfide as an attractant and coattractant for male and female codling moth (Lepidoptera: Tortricidae). Environ. Entomol..

[15] Jaffe B.D., Guédot C., Landolt P.J. (2018). Mass-trapping codling moth, *Cydia pomonella* (Lepidopteran: Torticidae), using a kairomone lure reduces fruit damage in commercial apple orchards. J. Econ. Entomol..

[16] Light D.M., Knight A.L., Henrick C.A., Rajapaska D., Lingren B., Dickens J.C., Reynolds K.M., Buttery R.G., Merrill G., Roitman J. (2001). A pear-derived kairomone with pheromonal potency that attracts male and female codling moth, *Cydia pomonella* (L.). Sci. Nat..

[17] Witzgall P., Stelinski L., Gut L., Thomson D. (2008). Codling moth management and chemical ecology. Annu. Rev. Entomol..

[18] Landolt P.J., Brumley J.A., Smithhisler C.L., Biddick L.L., Hofstetter R.W. (2000). Apple fruit infested with codling moth are more attractive to neonate codling moth larvae and possess increased amounts of (E,E)-α-Farnesene. J. Chem. Ecol..

[19] Hern A., Dorn S. (2002). Induction of volatile emissions from ripening apple fruits infested with *Cydia pomonella* and the attraction of adult females. Entomol. Exp. Appl..

[20] Coracini M., Bengtsson M., Liblikas I., Witzgall P. (2004). Attraction of codling moth males to apple volatiles. Entomol. Exp. Appl..

[21] Natale D., Mattiacci L., Hern A., Pasqualini E., Dorn S. (2003). Response of female *Cydia molesta* (Lepidoptera: Tortricidae) to plant derived volatiles. Bull. Entomol. Res..

[22] Natale D., Mattiacci L., Pasqualini E., Dorn S. (2004). Apple and peach fruit volatiles and the apple constituent butyl hexanoate attract female oriental fruit moth, *Cydia molesta*, in the laboratory. J. Appl. Entomol..

[23] Piñero J.C., Dorn S. (2007). Synergism between aromatic compounds and green leaf volatiles derived from the host plant underlies female attraction in the oriental fruit moth. Entomol. Exp. Appl..

[24] Il’ichev A.L., Kugimiya S., Williams D.G., Takabayashi J. (2009). Volatile compounds from young peach shoots attract males of oriental fruit moth in the field. J. Plant Interact..

[25] Varela N., Avilla J., Gemeno C., Anton S. (2011). Ordinary glomeruli in the antennal lobe of male and female tortricid moth *Grapholita molesta* (Busck) (Lepidoptera: Tortricidae) process sex pheromone and host-plant volatiles. J. Exp. Biol..

[26] Najar-Rodriguez A., Orschel B., Dorn S. (2013). Season-long volatile emissions from peach and pear trees in situ, overlapping profiles, and olfactory attraction of an oligophagous fruit moth in the laboratory. J. Chem. Ecol..

[27] Joshi N.K., Hull L.A., Rajotte E.G., Krawczyk G., Bohnenblust E. (2011). Evaluating sex-pheromone-and kairomone-based lures for attracting codling moth adults in mating disruption versus conventionally managed apple orchards in Pennsylvania. Pest Manag. Sci..

[28] Landolt P.J., Suckling D.M., Judd G.J.R. (2007). Positive interaction of a feeding attractant and a host kairomone for trapping the codling moth, *Cydia pomonella* (L.). J. Chem. Ecol..

[29] Knight A.L., Light D.M., Trimble R.M. (2011). Identifying (E)-4, 8-Dimethyl-1, 3, 7-Nonatriene plus acetic acid as a new lure for male and female codling moth (Lepidoptera: Tortricidae). Environ. Entomol..

[30] Lu P.F., Huang L.Q., Wang C.Z. (2012). Identification and field evaluation of pear fruit volatiles attractive to the oriental fruit moth, *Cydia molesta*. J. Chem. Ecol..

[31] Barros-Parada W., Ammagarahalli B., Basoalto E., Fuentes-Contreras E., Gemeno C. (2018). Captures of oriental fruit moth, *Grapholita molesta* (Lepidoptera: Tortricidae), in traps baited with host-plant volatiles in Chile. Appl. Entomol. Zool..

[32] Knight A.L., Basoalto E., Hilton R., Molinari F., Zoller B., Hansen R., Krawczyk G., Hull L. (2013). Monitoring oriental fruit moth (Lepidoptera: Tortricidae) with the Ajar bait trap in orchards under mating disruption. J. Appl. Entomol..

[33] Cichon L., Fuentes-Contreras E., Garrido S., Lago J., Barros-Parada W., Basoalto E., Hilton H., Knight A.L. (2013). Monitoring oriental fruit moth (Lepidoptera: Tortricidae) with sticky traps baited with terpinyl acetate and sex pheromone. J. Appl. Entomol..

[34] Knight A.L., Hilton R., Basoalto E., Stelinski L.L. (2014). Use of glacial acetic acid to enhance bisexual monitoring of tortricid pests with kairomone lures in pome fruits. Environ. Entomol..

[35] Mujica V., Preti M., Basoalto E., Cichon L., Fuentes-Contreras E., Barros-Parada W., Krawczyk G., Nunes M.Z., Walgenbach J.F., Hansen R. (2018). Improved monitoring of oriental fruit moth (Lepidoptera: Tortricidae) with terpinyl acetate plus acetic acid membrane lures. J. Appl. Entomol..

[36] Padilha A.C., Arioli C.J., Boff M.I.C., Rosa J.M., Botton M. (2018). Traps and baits for luring *Grapholita molesta* (Busck) adults in mating disruption-treated apple orchards. Neotrop. Entomol..

[37] Kong W.N., Wang Y., Guo Y.F., Chai X.H., Li J., Ma R.Y. (2020). Behavioral effects of different attractants on adult male and female oriental fruit moths, *Grapholita molesta*. Pest Manag. Sci..

[38] Knight A.L., El-Sayed A.M., Judd G.J.R., Basoalto E. (2017). Development of 2-phenylethanol plus acetic acid lures to monitor obliquebanded leafroller (Lepidoptera: Tortricidae) under mating disruption. J. Appl. Entomol..

[39] El-Sayed A.M., Knight A.L., Basoalto E., Suckling D.M. (2018). Caterpillar-induced plant volatiles attract conspecific herbivores and a generalist predator. J. Appl. Entomol..

[40] Judd G.J., Knight A.L., El-Sayed A.M. (2017). Trapping *Pandemis limitata* (Lepidoptera: Tortricidae) moths with mixtures of acetic acid, caterpillar-induced apple-leaf volatiles, and sex pheromone. Can. Entomol..

[41] Preti M., Knight A.L., Favaro R., Basoalto E., Tasin M., Angeli S. (2021). Comparison of new kairomone-based lures for *Cydia pomonella* (Lepidoptera: Tortricidae) in Italy and USA. Insects.

[42] Löfstedt C. (1990). Population variation and genetic control of pheromone communication systems in moths. Entomol. Exp. Appl..

[43] Ioriatti C., Molinari F., Pasqualini E., De Cristofaro A., Schmidt S., Espinha I. (2003). The plant volatile attractant (E, Z)-2, 4-Ethyl-Decadienoate (DA2313) for codling moth monitoring. Bull. Zool. Agric..

[44] Trimble R.M., El-Sayed A.M. (2005). Potential of ethyl (2E, 4Z)-2, 4-decadienoate for monitoring activity of codling moth (Lepidoptera: Tortricidae) in eastern North American apple orchards. Can. Entomol..

[45] Meraner A., Brandstätter A., Thaler R., Aray B., Unterlechner M., Niederstätter H., Parson W., Zelger R., Dalla Via J., Dallinger R. (2008). Molecular phylogeny and population structure of the codling moth (*Cydia pomonella*) in Central Europe: I. Ancient clade splitting revealed by mitochondrial haplotype markers. Mol. Phylogenetics Evol..

[46] Mitchell V.J., Manning L.-A., Cole L., Suckling D.M., El-Sayed A.M. (2008). Efficacy of the pear ester as a monitoring tool for codling moth *Cydia pomonella* (Lepidoptera: Tortricidae) in New Zealand Apple Orchards. Pest Manag. Sci..

[47] Dekker T., Kárpáti Z., Ishikawa Y. (2020). Coding and evolution of pheromone preference in moths. Insect Sex Pheromone Research and Beyond. Entomology Monographs.

[48] Piñero J.C., Giovanni Galizia C., Dorn S. (2008). Synergistic behavioral responses of female oriental fruit moths (Lepidoptera: Tortricidae) to synthetic host plant-derived mixtures are mirrored by odor-evoked calcium activity in their antennal lobes. J. Insect Physiol..

[49] Myers C.T., Krawczyk G., Agnello A.M. (2009). Response of tortricid moths and non-target insects to pheromone trap color in commercial apple orchards. J. Entomol. Sci..

[50] Fuková I., Neven L.G., Bárcenas N.M., Gund N.A., Dalíková M., Marec F. (2009). Rapid assessment of the sex of codling moth *Cydia pomonella* (Linnaeus) (Lepidoptera: Tortricidae) Eggs and Larvae. J. Appl. Entomol..

[51] Shang S. (2021). Morphological characteristics of reproductive system of the codling moth *Cydia pomonella*. ARTOAJ.

[52] Schumacher P., WEyENETH A., Weber D.C., Dorn S. (1997). Long flights in *Cydia pomonella* L. (Lepidoptera: Tortricidae) measured by a flight mill: Influence of sex, mated status and age. Physiol. Entomol..

[53] Boivin T., Chadœuf J., Bouvier J., Beslay D., Sauphanor B. (2005). Modelling the interactions between phenology and insecticide resistance genes in the codling moth *Cydia pomonella*. Pest Manag. Sci..

[54] Gu H., Hughes J., Dorn S. (2006). Trade-off between mobility and fitness in *Cydia pomonella* L.(Lepidoptera: Tortricidae). Ecol. Entomol..

[55] Timm A.E., Geertsema H., Warnich L. (2006). Gene flow among *Cydia pomonella* (Lepidoptera: Tortricidae) geographic and host populations in South Africa. J. Econ. Entomol..

[56] Fieser L.F., Fieser M. (1944). Organic Chemistry.

[57] Opgrande J.L., Dobratz C.J., Brown E., Liang J., Conn G.S., Shelton F.J., With J. (2000). Benzaldehyde. Kirk-Othmer Encyclopedia of Chemical Technology.

[58] Haynes K.F., Zhao J.Z., Latif A. (1991). Identification of floral compounds from *Abelia grandiflora* that stimulate upwind flight in cabbage looper moths. J. Chem. Ecol..

[59] Honda K., Ômura H., Hayashi N. (1998). Identification of floral volatiles from *Ligustrum Japonicum* that stimulate flower-visiting by cabbage butterfly, *Pieris Rapae*. J. Chem. Ecol..

[60] Bruce T.J., Cork A. (2001). Electrophysiological and behavioral responses of female *Helicoverpa armigera* to compounds identified in flowers of African marigold, *Tagetes erecta*. J. Chem. Ecol..

[61] Piñero J.C., Prokopy R.J. (2003). Field Evaluation of plant odor and pheromonal combinations for attracting plum curculios. J. Chem. Ecol..

[62] Lohonyai Z., Vuts J., Kárpáti Z., Koczor S., Domingue M.J., Fail J., Birkett M.A., Tóth M., Imrei Z. (2019). Benzaldehyde: An alfalfa-related compound for the spring attraction of the pest weevil *Sitona humeralis* (Coleoptera: Curculionidae). Pest Manag. Sci..

[63] Ethington M.W., Hughes G.P., VanDerLaan N.R., Ginzel M.D. (2021). Chemically-mediated colonization of black cherry by the peach bark beetle, *Phloeotribus liminaris*. J. Chem. Ecol..

[64] Xiang H.-M., Ma R.-Y., Diao H.-L., Li X.-W., He X.-J., Guo Y.-F. (2017). Peach-specific aldehyde nonanal attracts female oriental fruit moths, *Grapholita molesta* (Lepidoptera: Tortricidae). J. Asia Pac. Entomol..

[65] Vallat A., Dorn S. (2005). Changes in volatile emissions from apple trees and associated response of adult female codling moths over the fruit-growing season. J. Agric. Food Chem..

[66] Buchbauer G., Jirovetz L., Wasicky M., Nikiforov A. (1993). Headspace and essential oil analysis of apple flowers. J. Agric. Food Chem..

[67] Knight A.L., Cichon L., Lago J., Fuentes-Contreras E., Barros-Parada W., Hull L., Krawczyk G., Zoller B., Hansen R., Basoalto E. (2014). Monitoring oriental fruit moth and codling moth (Lepidoptera: Tortricidae) with combinations of pheromones and kairomones. J. Appl. Entomol..

[68] Allred D.B. (1995). Responses of Males to a Pheromone Blend of Female Oriental Fruit Moth with and without *E*8, *E*10-Dodecadien-1-ol, a Pheromone Component of Codling Moth (Lepidoptera: Tortricidae). Doctoral Dissertation.

[69] Knight A.L., Basoalto E., Stelinski L.L. (2016). Variability in the efficacy of sex pheromone lures for monitoring oriental fruit moth (Lepidoptera: Tortricidae). J. Appl. Entomol..

[70] Zhang G., Chen J., Yu H., Tian X., Wu J. (2018). Molecular and functional characterization of pheromone binding protein 1 from the oriental fruit moth, *Grapholita molesta* (Busck). Sci. Rep..

