# Supplementary material for: Male and Female Tortricid Moth Response to Non-Pheromonal Semiochemicals
Source: Insects. 2023 Nov 16;14(11):884. doi: 10.3390/insects14110884 (PMC10671916; doi:10.3390/insects14110884)
Supplement: Supplementary file 1 [file insects-14-00884-s001.zip › insects-2698434-supplementary.pdf]

*insects*

MDPI

**SUPPLEMENTAL FIGURES 1-4**

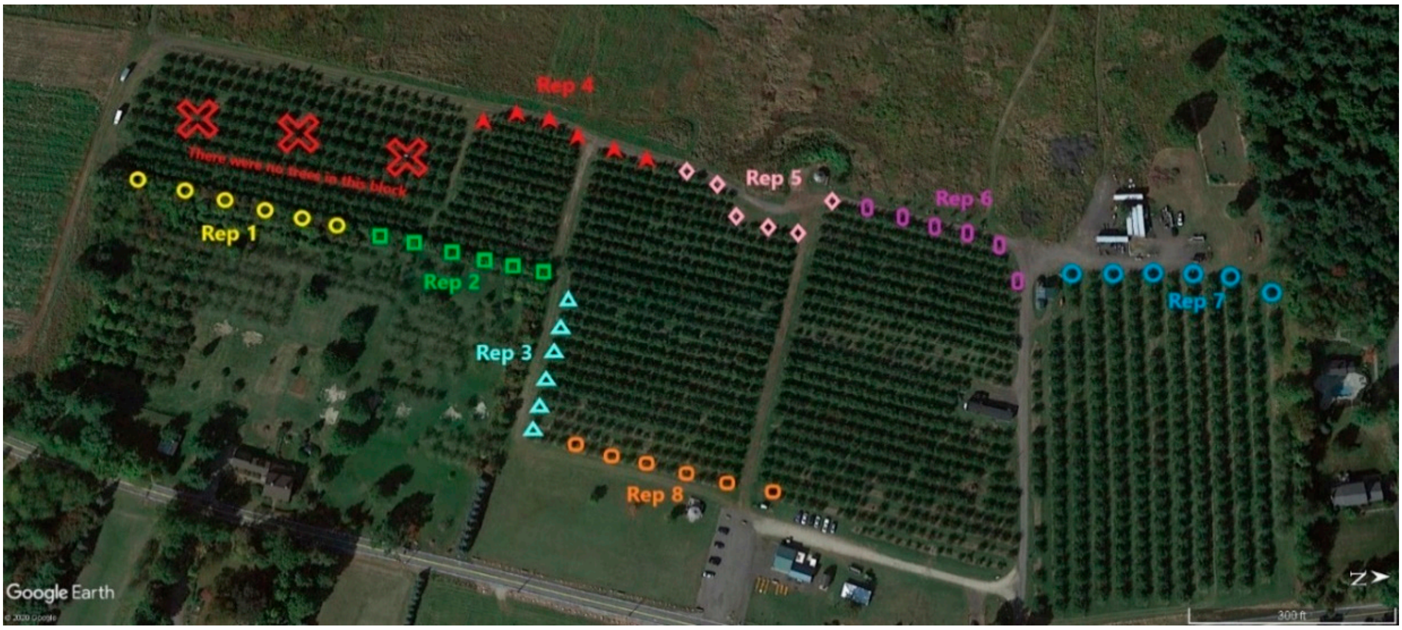


**Supplemental Figure S1.** Trap deployment along the perimeter of Sholan Farms in 2020.

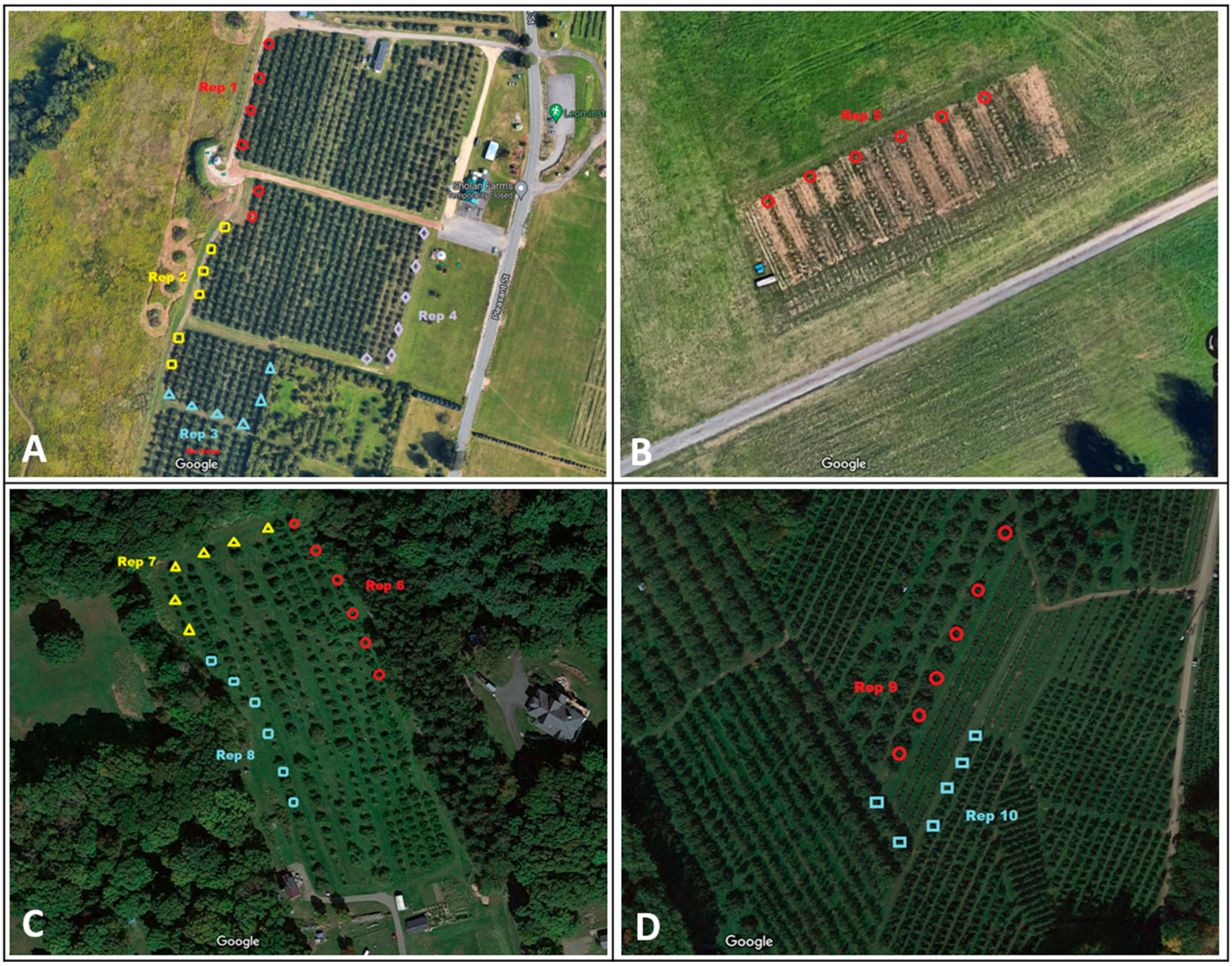


**Supplemental Figure S2.** Trap deployment at (**A**) Sholan farms, (**B**) UMass Agricultural Learning Center, (**C**) Sentinel Farm, and (**D**) Honeypot Hill Orchards in 2021.

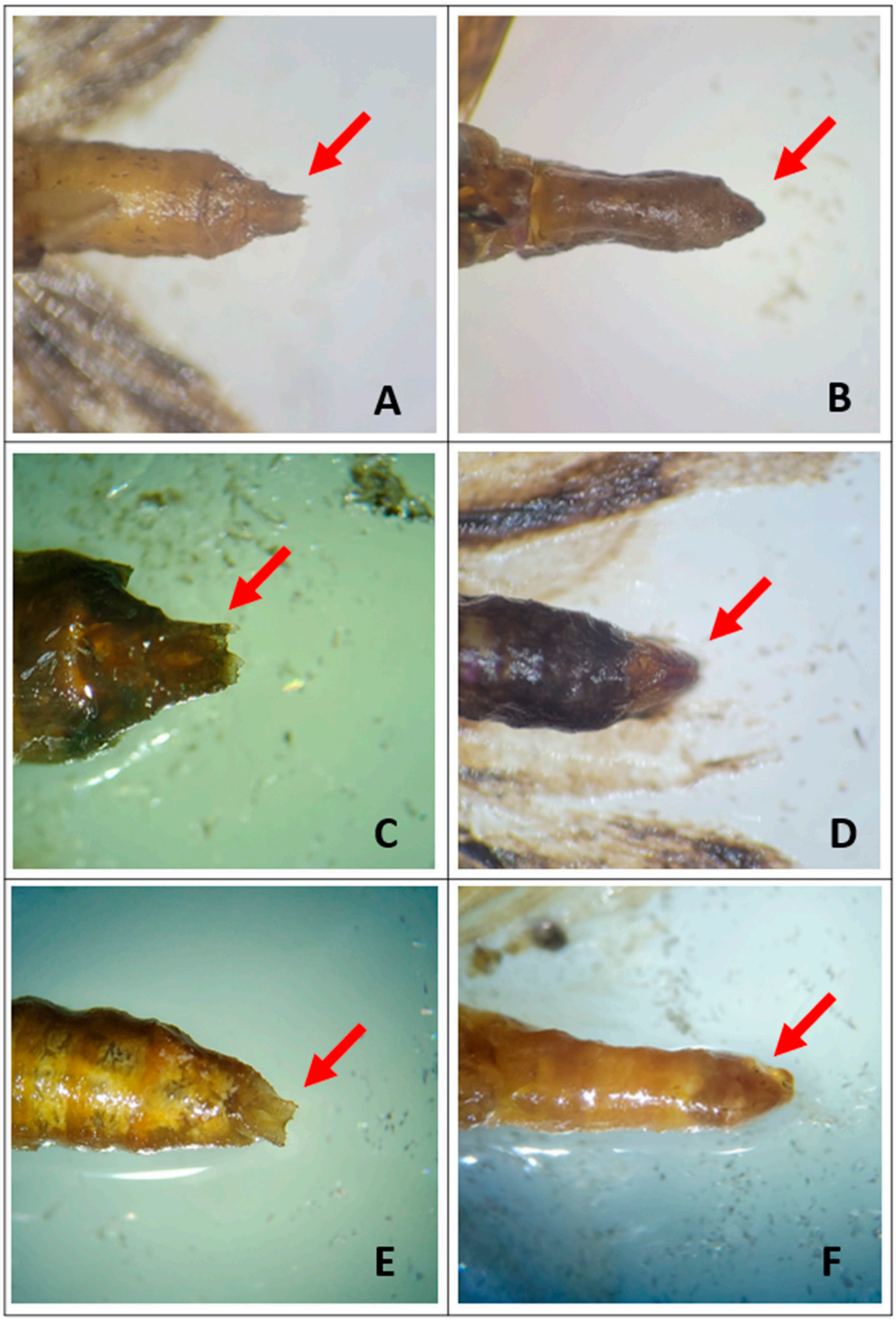


**Supplemental Figure S3.** Male genitalia (right) and female genitalia (left) of OFM (**A**,**B**), CM (**C**,**D**) and RBLR (**E**,**F**).

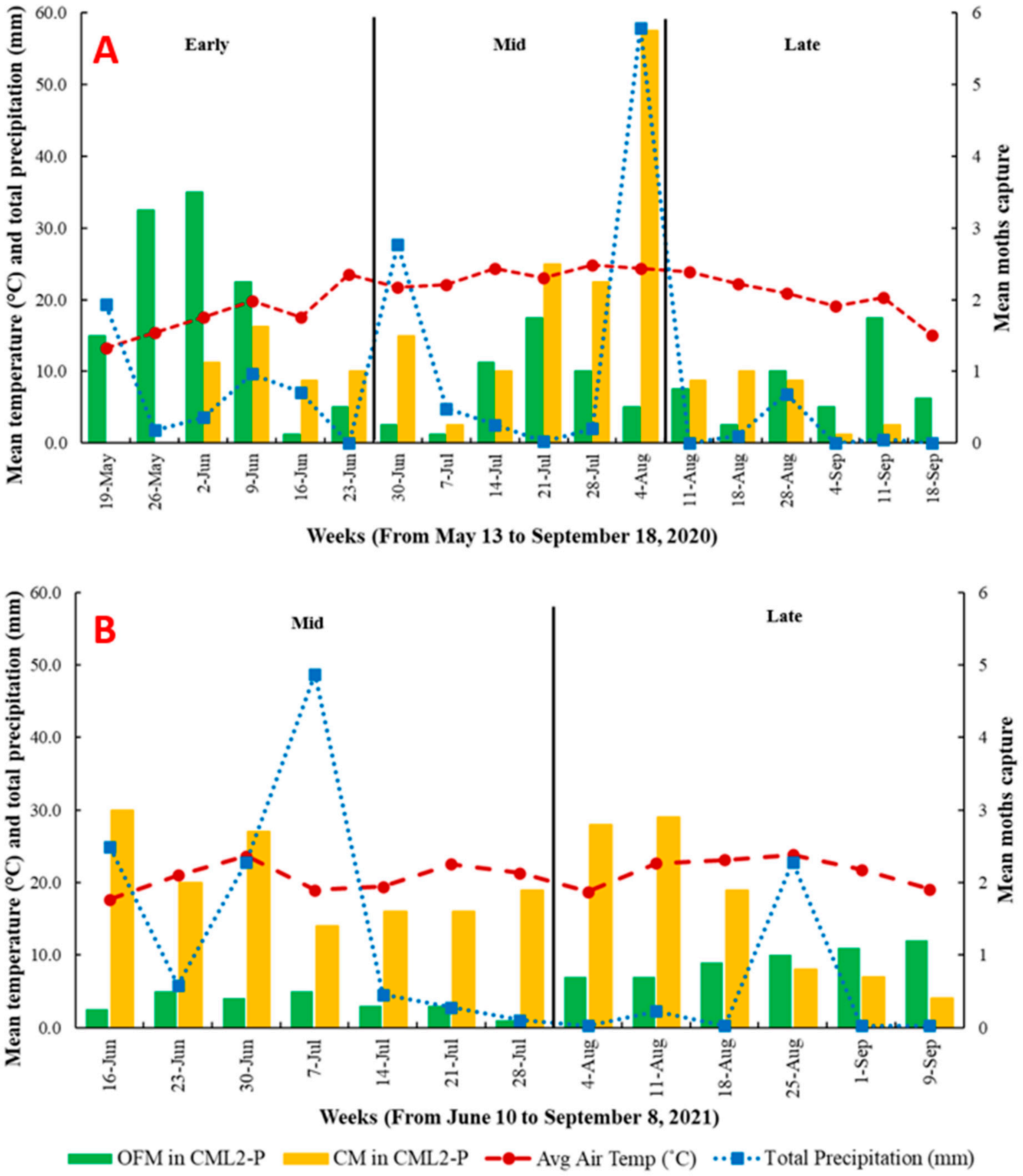


**Supplemental Figure S4.** Mean weekly captures of OFM (green bars) and CM (yellow bars) in early, mid, and late season during 2020 (**A**) and mid and late season during 2021 (**B**). Moth capture data correspond to the treatment with the pheromonal lure, CM L2-P. Mean temperature (°C) (red dotted lines) and total precipitation (mm) (blue dotted lines) were obtained from the Cornell University NEWA site using weather station data from Sholan Farms, Leominster, MA.
